# Unveiling patient profiles associated with elevated Lp(a) through an unbiased clustering analysis

**DOI:** 10.3389/fcvm.2025.1546351

**Published:** 2025-03-20

**Authors:** Miguel Saraiva, Jonatas Garcez, Beatriz Tavares da Silva, Inês Poças Ferreira, José Carlos Oliveira, Isabel Palma

**Affiliations:** ^1^ Department of Endocrinology, Diabetes and Metabolism, Local Health Unit of Santo António, Porto, Portugal; ^2^ Department of Clinical Chemistry, Local Health Unit of Santo António, Porto, Portugal

**Keywords:** lipoprotein(a) levels, clustering analysis, patient profiling, unsupervised learning, cardiovascular risk assessment

## Abstract

**Introduction:**

Lipoprotein(a) [Lp(a)] has been recognized as key factor in cardiovascular research. This study aimed to identify key patient profiles based on the characteristics of a Portuguese cohort of adults who were referred for Lp(a) measurement.

**Method:**

An unsupervised clustering analysis was performed on 661 Portuguese adults to identify patient profiles associated with lipoprotein a [Lp(a)] based on a range of demographic and clinical indicators. Lp(a) levels were deliberately excluded from the algorithm, to ensure an unbiased cluster formation.

**Results:**

The analysis revealed two distinct clusters based on Lp(a) levels. Cluster 1 (*n* = 336) exhibited significantly higher median Lp(a) levels than Cluster 2 (*n* = 325; *p* = 0.004), with 46.4% of individuals exceeding the 75 nmol/L (30 mg/dl) risk threshold (*p* < 0.001). This group was characterized by older age (median 57 vs. 45 years), lower body mass index (27.17 vs. 29.40), and a majority male composition (73.8% vs. 26.5%). Additionally, Cluster 1 displayed a higher prevalence of hypertension (56.5% vs. 31.1%), diabetes *mellitus* (38.7% vs. 17.2%), and dyslipidemia (88.7% vs. 55.4%). These data suggest that the Cluster 1 profile has a potential increased risk for cardiovascular complications and underscore the importance of considering specific patient profiles for Lp(a) screening and cardiovascular risk assessment.

**Conclusion:**

Despite the study limitations, including single-institution data and potential selection bias, this study highlights the utility of cluster analysis in identifying clinically meaningful patient profiles and suggests that proactive screening and management of Lp(a) levels, particularly in patients with characteristics resembling those of Cluster 1, may be beneficial.

## Introduction

Lipoprotein(a) [Lp(a)] – an apolipoprotein B-containing lipoprotein that structurally resembles low-density lipoprotein (LDL) with the specific apolipoprotein(a) (1, 2) – has become a focal point in cardiovascular research. Earlier evidence from Mendelian randomization and large cohort studies suggested an association between Lp(a) and atherosclerotic cardiovascular disease (ASCVD), underscoring its significance in the pathophysiology of cardiovascular diseases (CVD) and sparking increased interest in this lipoprotein (3, 4). Elevated Lp(a) levels, defined as exceeding 75 nmol/L (30 mg/dl), have consistently and independently been associated to an increased risk of ASCVD (2), coronary heart disease (5), myocardial infarction (6), and stroke (7). Of note, the prevalence of elevated Lp(a) levels in European populations varies significantly, ranging from 7% to 36% (8).

**Figure 1:**
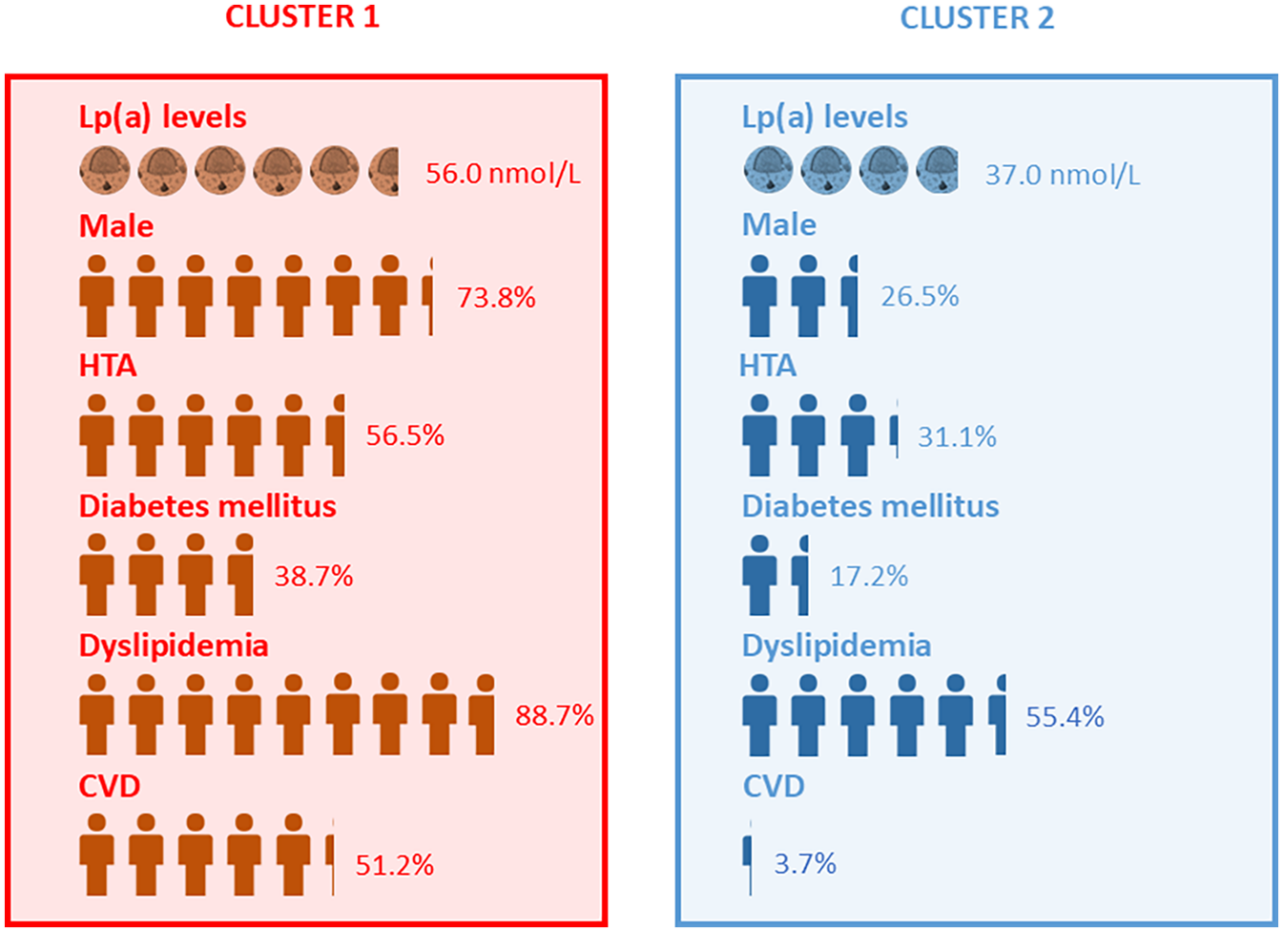
Cluster 1 and Cluster 2 characterization regarding the most relevant parameters.

Beyond its pivotal role in atherosclerosis, Lp(a) exerts systemic effects. High levels of Lp(a) have been correlated with chronic kidney disease (9), liver disease and inflammation (10), while paradoxically, low levels have been observed in diabetes *mellitus* (DM) patients (11), despite DM being a recognized CVD risk factor (11, 12). These findings highlight the multifaceted involvement of Lp(a) in systemic health, challenging our understanding of its multifunctional role in disease mechanisms.

Genetic determinants account for approximately 90% of the variation in Lp(a) levels, underscoring a tendency for familial clustering of elevated levels (13, 14). Such familial aggregation suggests an increased risk of CVD among family members, given the established association between elevated Lp(a) levels and cardiovascular risk.

The K-means algorithm, grounded in clustering theory, identifies inherent patterns within a dataset by dividing it into distinct groups (clusters), which aggregate similar items. This tool was previously proved valuable in distinguishing complex patient profiles, enhancing the understanding of various health conditions (15, 16). In the context of our research, K-means clustering was applied to the demographic and clinical data of a sample of patients; after forming the clusters, Lp(a) values were assessed within each group.

Leveraging this analytical framework, this study aims to unravel patient profiles of interest, based on the attributes of a Portuguese sample of adult individuals who were referred to Lp(a) measurement. By pinpointing the specific attributes consistently associated with elevated Lp(a) levels, we intend to refine diagnostic strategies, enabling clinicians to tailor CVD risk management more precisely and recommend Lp(a) screening for individuals exhibiting particular risk profiles.

## Method

This cross-sectional and retrospective study focused on adult individuals (≥18 years) who were hospitalized or under medical care at the *Centro Hospitalar Universitário de Santo António*, in Portugal, and had undergone a serum Lp(a) analysis. Data was collected from hospital medical records between August 2018 and June 2022. All serum Lp(a) levels were quantified by an immunoturbidimetric assay (Roche Diagnosis, Basilea, Swiss) at *Laboratório de Química Analítica do Centro Hospitalar Universitário de Santo António*, ensuring precision and minimizing potential bias. To identify patterns within demographic and clinical data, an unsupervised K-means clustering algorithm was employed, deliberately excluding Lp(a) levels to ensure an unbiased clustering formation. The algorithm considered a total of 14 variables, including age, sex, body mass index (BMI), hypertension (HTA), DM, dyslipidemia, ASCVD, family history of CVD, chronic renal disease, hypothyroidism, creatinine, triglycerides, LDL, and high-density lipoprotein (HDL) across a sample of 661 individuals. Initially, 26 variables were considered for this analysis. Of these, seven variables were excluded because they had ≥30% missing values (weight, height, menopause, HbA1c, oxidized LDL, ApoB and ultrasensitive CRP), two were excluded due to highly imbalanced class distributions, with one class representing less than 5% of the patients (chronic liver disease and acute inflammation), and three were excluded because they were highly correlated with other variables (Non-HDL, total cholesterol and VLDL), which would have introduced redundancy into the analysis. Patients were divided into clusters according to their similarities and then Lp(a) levels were evaluated within each cluster. Although lipid-lowering therapies were not included in the clustering analysis, they were analyzed within each cluster, including the proportion of patients taking statins (at various intensities), ezetimibe, and PCSK9 inhibitors.

The determination of the best number of clusters (*k* = 2) was based on the comparison of several quality scores (i.e., index scores available in the Nbclust package from R software. Variable selection consisted in three main steps. First, variables with a proportion of missing values ≥30% were excluded from the analysis; Second, unbalanced categorical variables, where one class represented <5% of individuals, were not considered. Third, correlations between numerical variables were assessed using Spearman's correlation test, with only one variable selected from pairs presenting correlation coefficients above 0.7.

Characterization of the obtained clusters involved a comprehensive descriptive analysis of demographic and clinical attributes, along with the Lp(a) levels of the corresponding individuals. Numerical variables were summarized using the median, 25th percentile, and 75th percentile, while categorical variables were presented as absolute and relative frequencies. Regarding inferential statistics, Mann–Whitney U tests were carried out to assess the association between the cluster formation and numerical variables of interest, and Chi-squared tests were applied to evaluate the association between the cluster formation and categorical variables. Bivariate analyses were performed to compare the obtained clusters. The significance level was set as 0.05, and *p*-values obtained through multiple comparisons were adjusted with the Benjamini-Hochberg correction.

## Results

The 661 individuals were allocated into two distinct clusters based on a comprehensive analysis of their demographic and clinical profiles. Table 1 details the characterization of each cluster, including their median Lp(a) levels, which were not included in the clustering analysis.

**Table 1:** Clusters characterization regarding Lp(a) levels, and demographic and clinical information of the corresponding individuals.

Variables	Total (*n* = 661)	Cluster 1 (*n* = 336)	Cluster 2 (*n* = 325)	*p*-value
Lp(a) levels (nmol/L)				
Med (P25; P75)	44.60 (13.00; 161.30)	56.00 (13.75; 195.85)	37.00 (12.60; 115.90)	**0**.**004**
≥75 nmol/L	263 (39.8%)	156 (46.4%)	107 (32.9%)	**0**.**001**
<75 nmol/L	398 (60.2%)	180 (53.6%)	218 (67.1%)	
≥125 nmol/L	203 (30.7%)	124 (36.9%)	79 (24.3%)	**<0**.**001**
<125 nmol/L	458 (69.3%)	212 (63.1%)	246 (75.7%)	
Patient characteristics				
Sociodemographics				
Age Med (P25; P75)	52.00 (42.00; 59.00)	57.00 (50.00; 63.00)	45.00 (36.00; 53.00)	**<0**.**001**
BMI Med (P25; P75)	28.08 (24.69; 32.60)	27.17 (24.69; 31.00)	29.40 (24.70; 35.90)	**<0**.**001**
Male *n* (%)	334 (50.5%)	248 (73.8%)	86 (26.5%)	**<0**.**001**
Comorbidities, *n* (%)				
HTA	291 (44.0%)	190 (56.5%)	101 (31.1%)	**<0**.**001**
Diabetes mellitus	186 (28.1%)	130 (38.7%)	56 (17.2%)	**<0**.**001**
Type 2 diabetes	150 (22.7%)	109 (32.4%)	41 (12.6%)	**<0**.**001**
Dyslipidemia	478 (72.3%)	298 (88.7%)	180 (55.4%)	**<0**.**001**
CVD	184 (27.8%)	172 (51.2%)	12 (3.7%)	**<0**.**001**
Family history of CVD	86 (13.0%)	51 (15.2%)	35 (10.8%)	0.117
Chronic renal disease	62 (9.4%)	57 (17.0%)	5 (1.5%)	**<0**.**001**
Hypothyroidism	43 (6.5%)	9 (2.7%)	34 (10.5%)	**<0**.**001**
Laboratorial data, Med (P25; P75)				
Creatinine (mg/ml)	0.82 (0.68; 1.00)	0.93 (0.81; 1.15)	0.71 (0.62; 0.84)	**<0**.**001**
Triglycerides (mg/ml)	106.00 (76.00; 159.00)	130.00 (89.75; 189.25)	92.00 (66.00; 126.00)	**<0**.**001**
HDL (mg/ml)	47.00 (39.00; 56.00)	42.00 (35.00; 49.00)	53.00 (44.00; 64.00)	**<0**.**001**
LDL (mg/ml)	89.00 (68.00; 118.00)	75.00 (57.00; 94.00)	107.00 (86.00; 143.00)	**<0**.**001**
Lipid lowering therapies, *n* (%)				
Statins				
Yes, high intensity	186 (28.3%)	154 (46.0%)	32 (9.9%)	**–**
Yes, moderate intensity	145 (22.0%)	98 (29.3%)	47 (14.6%)	**–**
Yes, low intensity	18 (2.7%)	11 (3.3%)	7 (2.2%)	**–**
No	309 (47.0%)	72 (21.5%)	237 (73.4%)	**–**
Missing	3	1	2	
Ezetimibe				
Yes	92 (14.0%)	76 (22.7%)	16 (4.9%)	**–**
No	567 (86.0%)	259 (77.3%)	308 (95.1%)	**–**
Missing	2	1	1	
iPCSK9				
Yes	1 (0.2%)	1 (0.3%)	0 (0%)	**–**
No	659 (99.8%)	334 (99.7%)	325 (100.0%)	**–**
Missing		1	0	

Bold values indicate *p*-values <0.05, representing statistically significant results.

Cluster 1 (*n* = 336) showed higher median Lp(a) levels compared to Cluster 2 (*n* = 325) (56.00 vs. 37.00 nmol/L, corresponding to approximately 33.96 and 26.05 mg/dl, respectively, *p* = 0.004). Moreover, a significant portion of Cluster 1 (46.4%) had Lp(a) levels ≥75 nmol/L (30 mg/dl) CVD risk threshold (*p* = 0.001), compared to 32.9% in Cluster 2. Additionally, the prevalence of very high levels of Lpa (≥125 nmol/L or ≥50 mg/dl) was also higher in Cluster 1 (36.9% vs. 24.3%, *p* < 0.001). Sociodemographic analysis indicated that Cluster 1 contained older individuals (median age: 57 vs. 46 years, *p* < 0.001) and a higher proportion of males (73.8% vs. 26.5%, *p* > 0.001).

From a clinical perspective, individuals in Cluster 1 had a lower BMI (27.17 vs. 29.40, *p* < 0.001). Cluster 1 also showed higher prevalence rates of HTA (56.5% vs. 31.1%, *p* > 0.001), DM (38.7% vs. 17.2%, *p* > 0.001), Type 2 DM (32.4% vs. 12.6, *p* < 0.001) and dyslipidemia (88.7% vs. 55.4%, *p* > 0.001). Remarkably, almost all patients with CVD or chronic renal disease are in Cluster 1 (93.5% and 91.9%, respectively), despite both clusters having similar distributions of patients with and without a family history of CVD (*p* = 0.117).

Laboratory data showed that Cluster 1 had higher levels of creatinine and triglycerides (*p* < 0.001), and lower levels of HDL and LDL than Cluster 2 (*p* < 0.001). Of note, the interpretation of LDL and HDL levels should be approached with caution due to the influence of lipid-lowering medications. In Cluster 1, a higher percentage of patients were taking lipid-lowering medications, specifically statins and ezetimibe (78.5% and 22.7%) compared to Cluster 2 (26.6% and 4.9%, *p* < 0.001), which likely contributes to the observed lower levels of LDL and HDL in Cluster 1. Total cholesterol was higher in Cluster 2 than Cluster 1 (184.00 vs. 146.00, *p* < 0.001), with a similar pattern observed for non-HDL (125.50 vs. 100.00, *p* < 0.001). Conversely, very-low-density lipoprotein (VLDL) levels were higher in Cluster 1 compared to Cluster 2 (24.00 vs. 18.00, *p* < 0.001) (data not shown).

When assessing the similarities and differences between the clusters and the overall population, several patterns emerge. Cluster 1, with its higher median age and higher prevalence of comorbidities such as HTA, DM, and dyslipidemia, appears to be more representative of a higher-risk demographic profile compared to the overall population. This is reflected by the elevated Lp(a) levels and the high percentage of patients exceeding the CVD risk threshold (≥75 nmol/L/30 mg/dl). Figure 1 visually delineates the most relevant attributes of Cluster 1 and Cluster 2, offering an illustrative summary of the results to facilitate the interpretation of the clinical profiles of each cluster.

## Discussion

This exploratory analysis provides a nuanced exploration of clinical profiles related to Lp(a) levels in a Portuguese adult cohort, revealing two distinct clusters with significant cardiovascular risk implications.

Cluster 1, characterized by higher levels of Lp(a), older age, lower BMI and a higher prevalence of CVD and related comorbidities (e.g., HTA and dyslipidemia), parallels with established literature linking elevated Lp(a) levels to increased CVD risk and common comorbidities (1, 3, 5, 6, 8). Interestingly, Cluster 1 has a higher proportion of men (73.8%), which contrasts with previous studies indicating that elevated Lp(a) levels are more frequently observed in women (1, 3). However, the difference in the number of males between clusters does not imply that men have higher Lp(a) concentrations. Since the clusters are defined by Lp(a) levels, this gender distribution difference warrants further investigation to better understand the complex relationship between gender and Lp(a) levels and cardiovascular risk.

A notable deviation from previous studies is the higher prevalence of DM in Cluster 1, challenging the documented inverse relationship between Lp(a) and DM (3). Still, while low levels of Lp(a) may be a risk factor for developing type 2 DM, elevated Lp(a) levels have been linked to the development of macro and microvascular complications, including CVD, coronary artery disease, nephropathy, and neuropathy (17). The finding that 94.3% of individuals in this cohort with previous cardiovascular events and type 2 DM fall into Cluster 1 accentuates the critical role of Lp(a) as a marker of recurrent cardiovascular events, as previously reported, independently of HbA_1c_ status (18). Furthermore, 78.6% of patients in Cluster 1 are on statin therapy, compared to only 26.7% in Cluster 2, reflecting the higher prevalence of dyslipidemia in this group. Given that statin therapy has been linked to an increased risk of developing type 2 DM, this effect may partially explain the higher DM prevalence observed in Cluster 1. However, it is important to note that the difference in DM prevalence between clusters does not necessarily imply a direct causal relationship between Lp(a) levels and DM risk.

A higher prevalence of creatinine levels in Cluster 1 also aligns with literature, which has suggested that impaired renal function can contribute to elevated Lp(a) levels (19, 20). While this study was not designed to establish causality, it is important to recognize the potential role of renal dysfunction in Lp(a) elevation within this cluster. Future research should further explore this relationship to better understand the underlying mechanisms.

In fact, patients with poor metabolic control tend to have higher Lp(a) levels, emphasizing the importance of metabolic control in managing cardiovascular risk factors in DM patients (17). The disparity in lipid profiles within Cluster 1, characterized by lower LDL and HDL cholesterol levels, could reflect the intensive lipid-lowering treatments in these individuals. This implies that individuals in this cluster were likely previously recognized as having more severe dyslipidemia, prompting proactive treatment approaches to avert potential cardiovascular events.

Interestingly, the homogeneous distribution of patients with a family history of CVD across both clusters suggests a more complex interaction between genetic factors and Lp(a) levels than previously expected (3). Given the genetic determination of Lp(a) levels, further research is warranted to explore this relationship and uncover additional patterns of clinical interest, especially among individuals with high LDL cholesterol levels (13).

In clinical practice, patients with a profile similar to cluster 1 – older male patients with HTA, DM, dyslipidemia and CVD – warrant closer monitoring of risk factors, including measurement of Lp(a) levels. Understanding the characteristics that typically accompany elevated Lp(a) is valuable for healthcare professionals to support the referral of Lp(a) measurement and for treatment guidance to mitigate patients' cardiovascular risk.

Although the majority of patients with elevated Lp(a) were in Cluster 1, there was still a substantial proportion (32.9%) with Lp(a) ≥ 75 nmol/L (30 mg/dl) who were allocated to Cluster 2. Regardless of Lp(a)'s recognized value as a predictive biomarker of cardiovascular risk (21), this observation aligns with the notion that Lp(a) levels are not the sole predictor of cardiovascular risk and that patient management should be personalized based on a combination of factors. Further studies to uncover additional patterns of clinical interest are required, particularly linking Lp(a) with family history of CVD.

This analysis builds on previous work by further delineating the complexity of cardiovascular risk in relation to Lp(a) levels. While previous work established the prevalence of elevated Lp(a) levels in the Portuguese cohort and its association with traditional CV risk factors, the current study extends these findings by identifying two distinct clusters of patients associated with different Lp(a) levels. Cluster 1, with higher Lp(a) and prevalent comorbidities, echoes previous observations and highlights the multifaceted nature of cardiovascular risk. Moreover, our findings suggest that Lp(a) could serve as a valuable marker not only for identifying patients at risk of cardiovascular events but also for detecting individuals who are at greater risk for recurrent events, such as those with DM, independently of HbA1c status. This has the potential to refine patient stratification and management strategies. Additionally, the distribution of family history of CVD across both clusters raises new questions about the role of genetic factors in influencing Lp(a) levels and their implications for cardiovascular risk. It is important to emphasize that while our analysis reveals significant associations, it does not establish a causal relationship between Lp(a) levels and the variables examined. Further research is needed to explore these complex interactions.

Despite the limitations inherent to the study, including potential selection bias and data sourced from a single institution, these results contribute to the evolving landscape of cardiovascular risk assessment. Selection bias may arise from the fact that our cohort consists of patients who underwent Lp(a) testing due to pre-existing conditions, such as dyslipidemia, potentially overrepresenting individuals at higher risk. Additionally, as single-center study, our findings may not be fully generalizable to broader populations. By delineating distinct patient clusters, this analysis highlights the multifactorial nature of cardiovascular risk and suggests the potential for more refined patient stratification.

In conclusion, this study underscores the potential of cluster analysis in identifying clinically significant patient profiles and suggests that proactive Lp(a) screening and management could be valuable, particularly for higher-risk individuals with a clinical profile similar to Cluster 1. Future studies should expand on these findings, incorporating broader datasets to validate and refine the proposed clinical profiles.

## Data Availability

The raw data supporting the conclusions of this article will be made available by the authors, without undue reservation.
